# The Development of Filler Morphology in Dental Resin Composites: A Review

**DOI:** 10.3390/ma14195612

**Published:** 2021-09-27

**Authors:** Jiani Liu, Hao Zhang, Huijun Sun, Yanru Liu, Wenlin Liu, Bo Su, Shibao Li

**Affiliations:** 1 State Key Laboratory of Military Stomatology and National Clinical Research Center for Oral Diseases, Department of Dental Materials, School of Stomatology, The Fourth Military Medical University, Xi’an 710032, China; Jenny170219@163.com (J.L.); ly_zhanghao@sina.cn (H.Z.); feier_22991@163.com (Y.L.); liuwenlin0903@163.com (W.L.); 2 The Affiliated Hospital of Stomatology, School of Stomatology, Zhejiang University School of Medicine, Key Laboratory of Oral Biomedical Research of Zhejiang Province, Hangzhou 310006, China; 3 Bristol Dental School, University of Bristol, Lower Maudlin Street, Bristol BS1 2LY, UK; iu20210@bristol.ac.uk (H.S.); B.Su@bristol.ac.uk (B.S.)

**Keywords:** dental resin composite, filler morphology, particulate fillers, fibrous fillers, novel-shaped fillers, mechanical properties, surface treatment

## Abstract

Dental resin composites (DRCs) with diverse fillers added are widely-used restorative materials to repair tooth defects. The addition of fillers brings an improvement in the mechanical properties of DRCs. In the past decade, diverse fillers have emerged. However, the change of emerging fillers mainly focuses on the chemical composition, while the morphologic characteristics changes are often ignored. The fillers with new morphologies not only have the advantages of traditional fillers (particles, fibrous filler, etc.), but also endow some additional functional characteristics (stronger bonding ability to resin matrix, polymerization resistance, and wear resistance, drug release control ability, etc.). Moreover, some new morphologies are closely related to the improvement of traditional fillers, porous filler vs. glass particles, core-sheath fibrous vs. fibrous, etc. Some other new morphology fillers are combinations of traditional fillers, UHA vs. HA particles and fibrous, tetrapod-like whisker vs. whisker and fibrous filler, mesoporous silica vs. porous and silica particles. In this review, we give an overall description and a preliminary summary of the fillers, as well as our perspectives on the future direction of the development of novel fillers for next-generation DRCs.

## 1. Introduction

Dental resin composites (DRCs) have become the most popular filling material in direct dental restorations while amalgam restorations have been gradually eliminated in clinical use due to negative effects, such as having a mismatched appearance with natural teeth, potential toxicity, and environmental pollution [1]. The excellent mechanical and aesthetic properties of DRCs help them meet the demands of daily chewing and aesthetic appearance in terms of tooth color [2,3]. In addition, with corresponding adhesive systems, the composites bond to tooth tissues without the need for extensive preparation of the dental cavity, avoiding the need for excessive removal of healthy dental tissues, which is common in the case of dental amalgam [4,5]. Thus, DRCs have attracted great interest in both scientific research and clinical practice.

DRCs have three main components: resin matrix which is a mixture of monomers (20–30 wt%); fillers(70–80 wt%); and a small amount of catalyst or initiator [6]. After curing, the resin matrix forms a three-dimensional network structure to encapsulate fillers. And fillers have usually been treated with a coupling agent to improve the bonding and stress transfer between fillers and the matrix [7]. Since DRC shrinkage after curing seems to result from resin matrix polymerization, much research has focused on the modification of the resin matrix. Various types of resin matrixes have been explored to attain specific properties, e.g., low polymerization shrinkage [8], antibacterial and/or fluoride release [9], and bio-safety [10]. While fillers, mainly acting as reinforcement, occupy the largest weight ratio in DRCs (about 70–80 wt%), they are surrounded by a cured resin matrix for preventing crack propagation in the event of fracture, changing the failure mode of the resin matrix after curing [11]. Fillers should have good stability and mechanical properties to ensure good final composites properties [2]. The application of fillers has already proved to be successful in many commercial and laboratory DRCs. DRCs are often named after the type of fillers, such as microfill DRCs, hybrid DRCs, micro-hybrid DRCs, and nanofill and nanohybrid DRCs, a convention adopted in the early days of their development [12,13,14].

Compared with the active research on resin matrixes, the research on fillers is far from sufficient, which may be because of the theory that shrinkage due to resin matrix polymerization is the main cause of failed DRC restorations. There is a preconception about fillers that their size, distribution, and chemical composition have more influence on the polishing and mechanical performance of DRCs and less on volume shrinkage [15]. A recent review on DRC fillers systematically discussed fillers based on their chemical composition and classification [16]. However, even with the same chemical composition, there are dramatic differences in the final properties of DRCs with different morphologies of fillers, especially for some novel-shaped fillers reported in recent years, such as core-sheath structure fillers and tetrapod-like fillers [17,18,19,20,21,22,23]. Therefore, it is insufficient to classify different forms of fillers and gauge trends in their development by only considering their chemical compositions. In addition, we found that fillers with certain special morphological features could reduce polymerization shrinkage through their spatial structure [24]. This may lead to an important breakthrough in the development of future DRCs. However, studies on filler morphologies are scattered across individual studies, and a systematic review of fillers based on morphology appears to be absent.

Here, we introduce a unique integrated classification based on filler morphologies to describe filler types and their development trends. Traditionally, the aspect ratio is an important parameter and one of the most important characteristics of fillers. Under this concept, we set the aspect ratio of 10 as a line of demarcation and classify fillers into two categories, i.e., particulate fillers (with an aspect ratio ≤ 10) and fibrous fillers (with an aspect ratio > 10). In addition, we propose a third category, i.e., novel-shaped fillers. This category contains either derivatives of traditional morphologies for which it is difficult to accurately determined their aspect ratios, such as bionic sea urchins, or some novel morphologies similar to traditional ones in appearance but completely different in microstructure, such as microspheres with mesoporous, hollow, or core-sheath microstructures.

## 2. Filler Type Classification by Morphology

**Figure 1:**
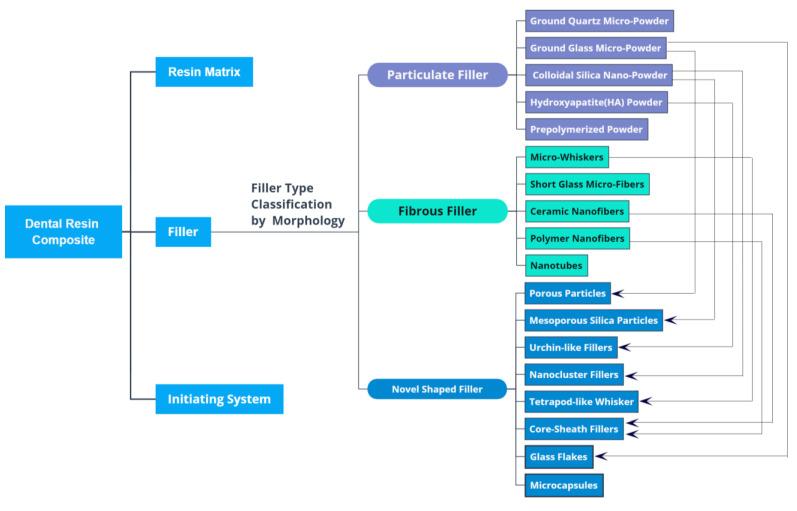
Classification of fillers in dental resin composites. The arrows illustrate the potential interconnections between traditional fillers and novel-shaped fillers.

Dimensionally, fillers can be classified into micro- and nano-scale fillers. Geometrically, they can be classified into particulate, fibrous, and novel-shaped fillers. Particulate fillers include ground quartz powder, ground glass powder, colloidal silica nanopowder, hydroxyapatite (HA) powder, and pre-polymerized powder. Fibrous fillers include microwhiskers, short glass microfiber, ceramic nanofiber, polymer nanofiber, and nanotube. Novel-shaped fillers include porous and mesoporous powder, urchin-like hydroxyapatite (UHA) powder, nanocluster powder, tetrapod-like whisker, core-sheath fiber, glass flake, and microcapsule. The classification of fillers is listed in Figure 1.

## 3. Particulate Fillers and Their Development

Particulate fillers most widely used in DRCs include ground quartz micropowder, ground glass micropowder, colloidal silica nanopowder, and pre-polymerized powder [25,26,27,28]. Although HA particles are used because of their bioactivity, they have been less frequently used in DRCs. The particulate fillers and details of their properties and application in commercial composites are listed in Table 1.

### 3.1. Ground Quartz Micropowder

Quartz is mainly composed of crystallized SiO_2_. Ground quartz micropowder has a large particle size (approximately 10–50 μm), rough surface, and irregular shape. DRC with a small portion of these fillers has superior mechanical properties to pure resin. When the filler content is increased to 65%, the DRC exhibits enhanced mechanical properties. However, the refractive index of quartz is higher than that of the resin matrix. This mismatch induces light scattering and allows less light to penetrate the deep layer. In addition, the DRC is hard to polish and prone to wear. 

### 3.2. Ground Glass Micropowder

Ground glass powder fillers, also known as alkaline glass fillers, have a smaller particle size (0.6–10 μm) than that of ground quartz powder. Though SiO_2_ is the main component, glass powder also contains Ba, Sr, and other elements [29]. These elements render composites with X-ray radiopacity without the need for additional radiopaque agents, which is beneficial for clinical diagnosis [16,30,31]. Some commercial DRCs, such as TetricEvoCeram (Ivoclar Vivadent, Schaan, Liechtenstein), Grandio (Voco, Cuxhaven, Germany), Esthet-x (Dentsply Caulk, Milford, DE, USA), and Herculite XRV (Kerr, Brea, CA, USA), contain this type of filler. Some DRCs have additional functionalities resulting from the addition of different types and concentrations of metallic elements to fillers [16], e.g., antibacterial silver [32]. In clinical application, when these DRCs are exposed to water or saliva in the oral environment, the metal ions may leach over time, inducing mixed effects on the DRC properties [33,34,35,36,37].

As DRCs tend to accumulate more bacterial biofilms and plaques, which is one of the main causes leading to restorative failure. Research has been carried out to specifically address secondary caries by developing novel DRCs with a biofilm-suppression ability and remineralization function for early caries recovery. Antimicrobial nanoparticles (e.g., Ag and ZnO) have been used in combination with other micropowders in DRCs [38].

### 3.3. Colloidal Silica Nanopowder

**Figure 2:**
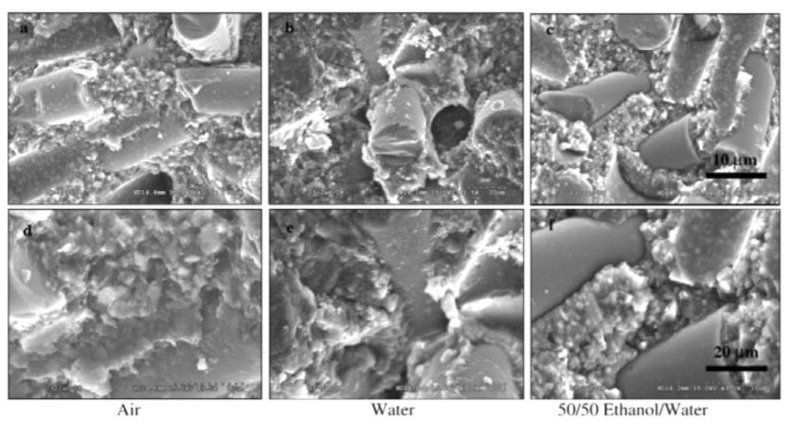
SEM micrographs of the fracture surface of short-glass-fiber-reinforced resin composites aged for 6 months in three different media: (**a**,**d**) air; (**b**,**e**) distilled water; and a (**c**,**f**) 50:50 (*v*/*v*) mixture of ethanol and distilled water. The images indicate the relatively large sizes of the fiber fillers compared with the surrounding particle fillers and the separation of the fiber fillers from the resin matrix in ethanol/water (**c**,**f**) compared with the other media. Images from [41].

Colloidal silica nano-powder was prepared using the nanotechnology referred to as the Stöber process [39,40]. This filler is much smaller (approximately 0.04–0.4 μm) than the abovementioned ground quartz micropowder and the ground glass micropowder. Most of these fillers are spheres with a lower surface roughness (Figure 2) [41], which improves the polishing property of DRCs. Nanosized SiO_2_ particles have been studied extensively and expanded to nanoclusters and mesoporous fillers. Due to the large specific surface area of nanosized SiO_2_ particles, the resin matrix viscosity dramatically increases and limits the content of nano-SiO_2_ in DRCs, leading to relatively poor mechanical properties if only one kind of this type of filler is used. In addition, nanosized SiO_2_ particles tend to agglomerate in the resin matrix because of their large specific surface area, thus forming stress concentrations in composites. Therefore, commercial DRCs filled with neat nanosized SiO_2_ particles are rare. Nanosized SiO_2_ particles have been extensively used in combination with other types of fillers (microparticles, fibrous fillers, whiskers, etc.), which can significantly improve the content of the filler, enhancing the mechanical properties [42,43,44] of DRCs and reducing polymerization shrinkage to a certain extent [24]. Alternatively, nanosized SiO_2_ particles can be used with other nanoparticles to form nanoclusters with reduced viscosity and enhance the properties of DRCs. They will be further discussed in Section 5.4.

### 3.4. Hydroxyapatite (HA) Powder

HA is an essential component of enamel and dentin. HA particles have low solubility at physiological pH [45]. When the pH is below 5.5, the dental hydroxyapatite undergoes dissolution, and some cariogenic bacteria are activated [46]. Hence, developing DRCs with remineralization capability will help reverse early caries. Nanoparticles of amorphous calcium phosphate, with a size of about 100 nm, have been incorporated into DRCs to gain better ion-release profiles due to the small size and increased surface area for chemical interactions [47]. This novel nanocomposite not only released high levels of Ca and P ions at low pH for remineralization but also demonstrated superior mechanical properties. HA powders with high aspect ratios have also been investigated [48,49,50,51,52,53], e.g., whiskers and fibers, but the dispersion and interfacial adhesion with the resin matrix remain unsatisfactory. In recent research, urchin-like HA exhibited stronger interfacial adhesion and improved mechanical properties of the resin composite than other types of HA fillers [54]. This will be discussed in more detail in Section 5.

### 3.5. Prepolymerized Powders

A pre-polymerized particle filler is produced by a multi-step process: ultra-fine filler is added to the resin matrix with mechanical mixing, followed by thermal polymerization and subsequent polymer grinding. This filler can significantly reduce the viscosity of the resin matrix as the filler loading level increases. In this DRC, there is no requirement that the pre-polymerized resin is the same as the final resin matrix [55]. The resultant composite has the advantage of low polymerization shrinkage and water absorption [56]. However, the flexural strength and modulus of the DRC are lower than those of hybrid composites [57].

## 4. Fibrous Fillers and Their Development

Compared to other types of fillers, the chemical compositions of fibrous fillers are different and feature a higher aspect ratio [58]. In terms of composition and aspect ratio, fibrous fillers can be divided into microwhiskers, shortglass microfiber, ceramic nanofiber, polymer nanofiber, and nanotube (Table 2). Among them, polymer nanofiber has the lowest rigidity. The whisker filler has relatively high rigidity and a low aspect ratio. Nanotube has a hollow tubular microstructure. Polymer and ceramic nanofibers have been extensively studied. The mechanical properties of DRCs can be considerably improved by the inclusion of only small amounts of fibrous fillers [59,60,61,62,63,64,65], which can strengthen and toughen DRCs through pinning, fiber pulling out, crack deflection, and bridging mechanisms. Other factors that influence the mechanical properties of the final DRCs are the content and orientation of the fibrous filler as well as the interfacial bonding between the fibrous filler and the resin matrix [62,66]. Generally, fibrous fillers are not added in large quantities due to their tendency for severe agglomeration, observed in microsized silicon carbide and silicon nitride whiskers [67,68], short glass fibers [65,69,70], and nanosized polymer and ceramic fibers [24,71].

### 4.1. Microwhisker

**Figure 3:**
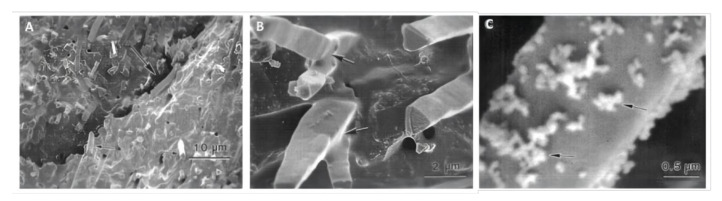
SEM micrographs of the fracture surfaces of the whisker composite at different ratios of whiskers to silica. (**A**) The composites containing silica-fused whiskers showed rougher fracture surfaces, with large fracture steps (large arrows) and pulled-out whiskers (small arrows). (**B**) The whiskers appeared to be well bonded with the matrix resin at whisker-resin interfaces, as indicated by the arrows. (**C**) The silica particles on the surface of the whiskers. Images from [68].

A whisker is a kind of filament grown in the form of a single crystal under natural or artificial conditions. Compared with other fillers, a whisker is defect-free, e.g., it lacks grain boundaries, dislocations, and holes. Its extraordinary strength is close to that of the theoretical value for a single crystal. Xu et al. [68] studied silicon nitride and silicon carbide whiskers and found that the type of whisker and the ratio of whiskers to silica significantly influenced the mechanical properties of the DRCs. These whiskers appeared to be well bonded with the resin matrix at whisker–resin interfaces (Figure 3A,B). The inclusion of silicon nitride whiskers also improved the strength and toughness of DRCs more than silicon carbide whiskers. Silicon carbide whiskers were capable of improving modulus and hardness compared to silicon nitride whiskers. When the ratio of silicon nitride whiskers/silica was 1:1, the DRC achieved the highest strength, of 246 ± 33 MPa; when the ratio of silicon carbide whiskers/silica was 5:1, the maximum strength of the DRC was 210 ± 14 MPa. In addition, by fusing dicalcium phosphate nanoparticles to the silicon carbide whisker’s surface (Figure 3C) [72], the final DRC released calcium and phosphorus ions and still maintained good mechanical performance with high flexural strength (167 MPa) [67].

### 4.2. Short Glass Microfiber

**Figure 4:**
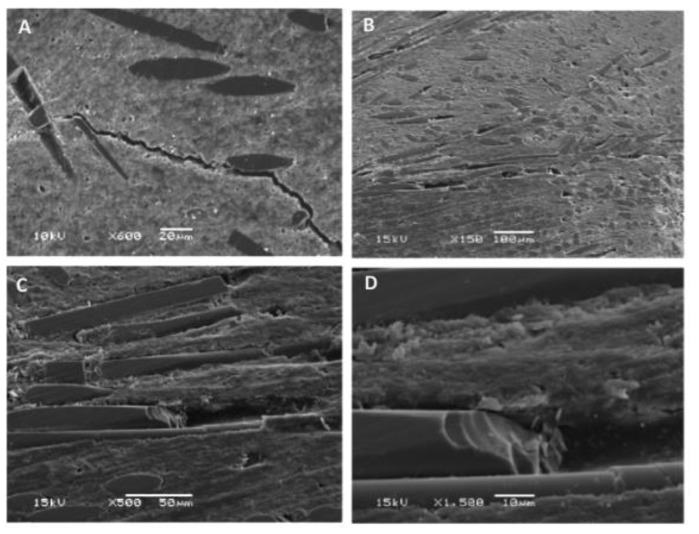
SEM micrographs of (**A**) a polished surface of a fiber composite with a propagating crack and (**B**–**D**) fracture surfaces with different magnifications showing fractured glass fibers Images from [65].

Short glass fiber is close to silica in chemical composition and has been widely used in DRCs. Usually, these fibers are more translucent than silicon-carbide-whisker-reinforced DRCs. Such DRCs used in clinics include Ever X Posterior (GC Dental, Tokyo, Japan), Restolux (Lee Pharmaceutical, South El Monte, CA, USA), and NovaPro^®^ Flow Flowable Composite (NovaPro, Mojave Court, CA, USA). They are usually used in high-load-bearing areas (especially in the posterior tooth fossa). Compared with DRCs with particulate fillers, short glass fibers have a better reinforcing effect in DRCs [73,74]. For example, compared with pure particulate resin composite Z250 (3M ESPE, St. Paul, MN, USA), Garoushi et al. [65] found that the inclusion of 22.5 wt% short glass fiber filler in the resin composite increased the flexural strength by nearly 99%, the compressive strength by 85%, and the fracture toughness by 300%. According to that study, the factor contributing to the reinforcing and toughening effects of short E-glass fibers in the DRC is the interpenetrating-polymer-network-polymer matrix (IPN-polymer matrix) structure (Figure 2) [41], which could include crack deflection, bridging, pinning, and other fracture energy absorption mechanisms (Figure 4) [65].

Lassila et al. [69] prepared a resin composite by mixing 27 wt% E-glass fiber (with micrometer- and millimeter-length scales) into 23 wt% dimethacrylate-based resin matrix and then adding 50 wt% silanized silica particulate filler. Compared with the conventional posterior DRC Filtek Supreme (3M ESPE, St. Paul, MN, USA), the fracture toughness and the flexural strength of this composite increased from 1.2 to 4.7 MPa·m^1/2^ and from 103.5 to 155 MPa, respectively. This again demonstrates that glass fiber has better reinforcing and toughening effects in a resin composite. However, the challenges of using glass fiber are water absorption and the leaching of soluble inorganic oxides, which aggravate the reduction in mechanical properties of glass-fiber-reinforced DRCs used in the oral environment.

### 4.3. Ceramic Nanofibers

**Figure 5:**
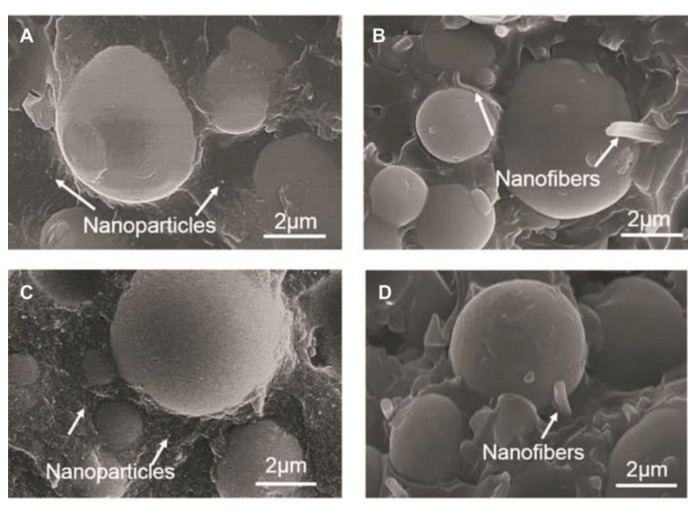
SEM micrographs of fractured surfaces of the Bis-GMA/TEGDMA composite reinforced with (**A**) 5 wt% SiO_2_ nanoparticles and 60 wt% microparticles;(**B**) 5 wt% SiO_2_ nanofibers and 60 wt% microparticles;(**C**) 10 wt% SiO_2_ nanoparticles and 60 wt% microparticles; and (**D**) 10 wt% SiO_2_ nanofibers and 60 wt% microparticles. Images from [24].

Compared to microsized shortglass fibers, nanosized ceramic fibers (SiO_2_, zirconia–yttria (ZY), and zirconia–yttria–silica (ZYS)) have a better reinforcing effect on DRCs when used in conjunction with other particulate fillers [24,75]. Particles were well dispersed in the interpenetrating polymer network (IPN) structure with nanofibers, as shown in Figure 5 [24]. Even a small amount of ceramic nanofibers was able to significantly improve the flexural strength, elastic modulus, and work of fracture of DRCs. In a study by Gao et al. [76], the flexural strength, elastic modulus, and work of fracture values of nanoglass-fiber-filled composites were increased by as much as 44%, 29%, and 66%, respectively. In another study [24], where the content of SiO_2_ nanofibers was 5 wt%, the flexural strength of the final DRC reached 118 MPa. Compared to SiO_2_ nanoparticles, zirconia–silica (ZS) and ZYS ceramic nanofibers showed better performance in improving the overall properties of DRCs, especially with improved wear resistance and reduced polymerization shrinkage [75]. The mechanical properties of DRCs with different contents of ZS or ZYS ceramic nanofibers were compared. When the DRCs contained 70 wt% glass particle fillers and 2.5 wt% ZS nanofibers or 5.0 wt% ZYS nanofibers, the DRCs exhibited the best performance in terms of flexural strength (FS), flexural modulus (FM), and energy at break (EAB). Nevertheless, the strengthening effect decreased when the content of ZS ceramic nanofibers was increased to 7.5 wt%, due to aggregation. This might be attributed to the stress concentration zone induced by nanofiber aggregates, leading to a weakened resin composite [77].

### 4.4. Polymer Nanofibers

Polymer nanofibers are usually used in engineering composites. Fong et al. studied DRCs reinforced with polymeric fibers [78]. Nylon-6 nanofibers were used and showed apparent reinforcement and toughening effects. When the content of polymer nanofibers was 5 wt%, the flexural strength and fracture toughness of the resin composite was 36% and 42% higher, respectively, than those of pure resin. However, the bonding interface between nylon-6 and the matrix required further optimization [24]. The hydrophilicity of nylon polymer induced water absorption, which reduced the strength of the nylon nanofiber itself as well as that of the composite.

### 4.5. Nanotubes

Nanotubes are hollow fibrous fillers with a high aspect ratio. They have the same strengthening and toughening mechanisms as fibrous fillers. Nanotubes vary in chemical composition, with the most widely studied being carbon nanotubes [79]. According to Zhang et al., silanized methacrylic groups on the surface of carbon nanotubes improved the flexural strength of the resin composite by 23%, from 115 to 142 MPa, albeit its appearance was un-aesthetically dark [80]. In two other separate studies, electrospunnylon-6/multilayer carbon nanotubes [81] and kaolinite nanotubes [82] were also used as reinforcements for the resin composite. However, the reinforcing effect of nanotubes in composites was strongly dependent on the content of nanotubes. When the loading of nanotubes exceeded 5%, the strengthening effect diminished. In another study on triclosan-encapsulated halloysite nanotubes (HNT/TCN), Cunha [83] found that the addition of 8 wt% HNT/TCN had a significant effect on the flexural strength of the composite, but the polymerization stress was higher than that of the composite without HNT, which might be connected with nanotube loading. When the mass fraction was increased to more than 10 wt%, the weakening instead of reinforcing effect was dominant.

## 5. Novel-Shaped Fillers and Their Development

Novel-shaped fillers include porous and mesoporous particles, urchin-like particles, nanoclusters, tetrapod-shaped whiskers, core-sheath fibers, flakes, and microcapsules (Table 3). Despite some being derived from pre-existing particulate and fibrous fillers, the morphology (e.g., urchin or tetrapod shape) and composition (dissimilar core-sheath materials) of novel-shaped fillers are beyond the boundaries of those for traditional fillers. Their surface either provides more mechanical chimerism (e.g., open-pore penetration or anchorage) or enhances the interfacial bonding (e.g., through more adhesive sheath).

### 5.1. Porous Particles

**Figure 6:**
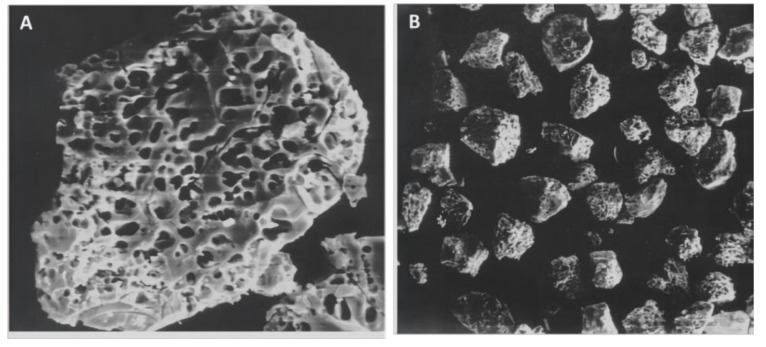
SEM micrographs of porous fillers after HF acid etching were shown at magnifications of (**A**) 2000× and (**B**) 500×. The liquid resin can enter the pores of the porous fillers, and the resulting strength effects are related to this phenomenon. Images from [86].

Depending on the pore size and shape, porosity, and chemical composition of the particles, porous particulate fillers can improve mechanical performance and drug-releasing [84,85]. The typical morphology of a porous filler is shown in Figure 6 [86].

Compared with traditional dense fillers, porous fillers can be penetrated by liquid resin, which can enhance the bonding strength between filler and resin matrix after crosslinking, thereby improving the mechanical properties of DRCs. In addition, the strong bonding between the porous filler and the resin matrix prevents the filler and the resin matrix from detaching, which can improve the wear resistance of DRCs [87]. Zandinejad et al. and Liu et al. [86,88] confirmed that adding porous glass-ceramic powder fillers improved the flexural strength and modulus of the DRCs but had less effect on tensile strength. However, the flexural strength did not change proportionally with the increasing porosity of the filler particles [88]. Porosity itself was not a determining factor in the mechanical strength of DRCs. The degree of resin penetration into the pores of porous fillers was another factor to be considered. If the resin matrix was incompletely infiltrated, the micromechanical coupling between porous fillers and the resin matrix was weak, which resulted in lower mechanical properties, especially flexural strength. A study by Ruddel et al. [61] validated this hypothesis. Two methods were investigated to address the resin infiltration problem. The first involved mixing porous fillers and resin under vacuum [89], and the second to add solvents to reduce resin viscosity and facilitate infiltration [90]. A novel DRC was proposed based on microfillers of anodic nanoporous alumina. This material exhibited better mechanical properties but seemed prone to aging, and the mechanical properties weakened dramatically when the DRC was loaded with drugs [85].

### 5.2. Mesoporous Silica Particles

**Figure 7:**
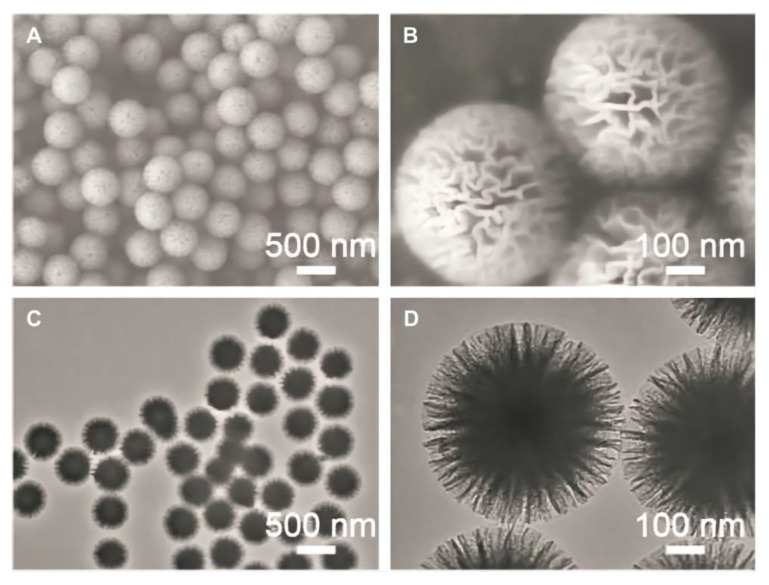
(**A**,**B**) Field emission scanning electron microscopy (FE-SEM) micrographs of wrinkled mesoporous silica and (**C**,**D**) TEM images of different magnifications of wrinkled mesoporous silica.Images from [91].

To control filler pore size and uniformity, mesoporous silica particles may be a better option. Wang et al. [91] introduced a novel porous filler, termed wrinkled mesoporous silica (WMS), which had a monodisperse spherical core with wrinkles extending radially outward from the center. Its rugged structure is shown in Figure 7 [91]. Similar to a porous filler, mechanical interlocking between resin matrix and WMS strengthened the DRC, improving its flexural strength, flexural modulus, compressive strength, and Vickers microhardness, compared to normal silica particles with the same diameter.

### 5.3. Urchin-like Fillers

**Figure 8:**
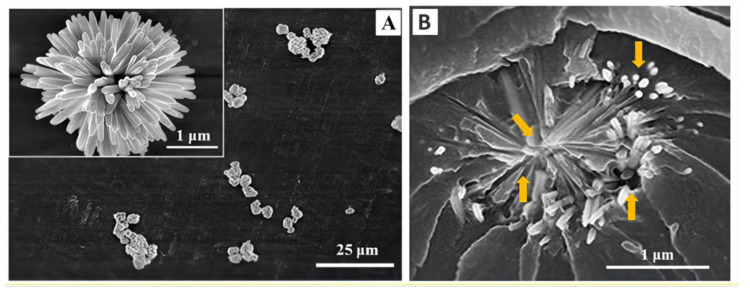
SEM micrographs of (**A**) UHA fillers and (**B**) the fracture surface of the dental resin with 20 wt%UHA. Images from [53].

In previous studies, HA whiskers or nanofibers were fabricated to improve the mechanical properties of DRCs [48,51]. Recently, a new type of urchin-like HA (UHA) powder combining the characteristics of both whiskers and fibers has emerged. This type of filler can be tightly embedded in the resin matrix, increasing the interfacial area and the bonding strength between the fillers and the matrix. In a study by Liu et al. [54], the enhancement effect of UHA was found to be better than that of irregular particle hydroxyapatite (IPHA) or hydroxyapatite whisker (HAW)-reinforced DRCs. It was found that with the increase in UHA content (20 and 30 wt%), the flexural modulus and microhardness of DRCs increased, but not strength. This might be attributed to the silanized UHA fillers being closely embedded in the matrix to form a strong cohesion between the resin matrix and fillers. The shape of the sea urchin might reduce aggregation in the resin matrix compared to IPHA and HAW fillers. The UHA particles were closely intertwined and did not affect the resin monomer movement, which improved resistance to breakage even under a high load. Integration of silanized UHA into DRCs containing silica nanoparticles could further improve the strength and modulus of DRCs. Figure 8 shows SEM images of UHA fillers (A) and 20 wt% UHA reinforced composite (B). Figure 8B shows a desirable bonding between UHA fillers and the matrix. No appreciable gaps or voids appeared on the fracture surface. In addition, UHA with an urchin-like structure was fully integrated into the resin matrix, forming strong interfacial bonding, which made the stress transfer more efficient between the UHA and the matrix.

### 5.4. Nanocluster Fillers

Nanoclusters are secondary particles created through the dense fusion of nanoparticles, typically SiO_2_ or ZrO_2_ or hybrid particles [92]. Several particles are fused together to form a large, covalently bonded cluster [93]. The silane coupling agent can penetrate gaps of the nanoclusters, improving the mechanical strength of the DRC [94,95].

**Figure 9:**
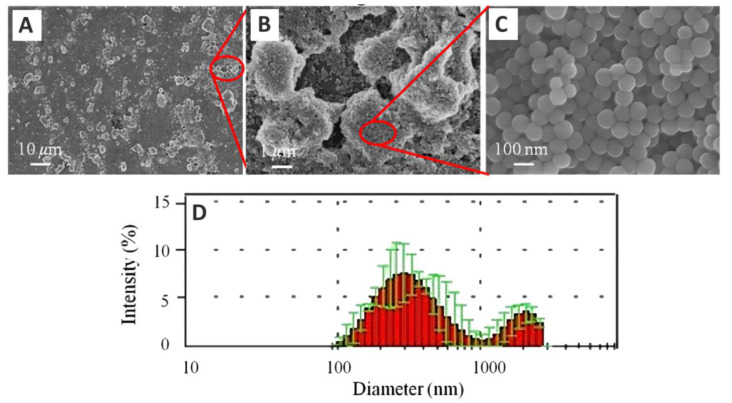
FE-SEM micrographs and particle size distributions of silica bimodal nanoclusters and magnified to varying degrees, indicated with scale bars of 10 μm (**A**), 1 μm (**B**) and 100 nm (**C**) at selected regions, and their size distribution (**D**), respectively.Images from [96].

Randolph et al. [17] revealed that Filtek Supreme XTE (3M ESPE, St. Paul, MN, USA) with nanoclusters exhibited better fracture strength than other commercial DRCs without nanoclusters. Nanoclusters in DRCs can absorb stress that promotes crack growth and, thus, fracture strength is improved. Novel bimodal SiO_2_ nanoclusters (as shown in Figure 9) were synthesized. The silica nanoclusters, namely, large, agglomerated particles consisting of numerous silica nanoparticles and silica aggregations with cavities, have a size distribution within the range of 0.07–2.70 μm and are shown in Figure 9 [96]. When the DRCs contained 70 wt% filler (50 wt% bimodal SiO_2_ nanocluster and 20 wt% unimodal SiO_2_), the flexural strength (104.8 ± 4.4 MPa), flexural modulus (6.2 ± 0.3 GPa), and compressive strength (205.8 ± 14.3 MPa) of the DRCs were 28%, 48%, and 42% higher, respectively, than those of the unimodal SiO_2_ (60 wt%) filled DRCs without bimodal SiO_2_ nanoclusters.

### 5.5. Tetrapod-like Whiskers

**Figure 10:**
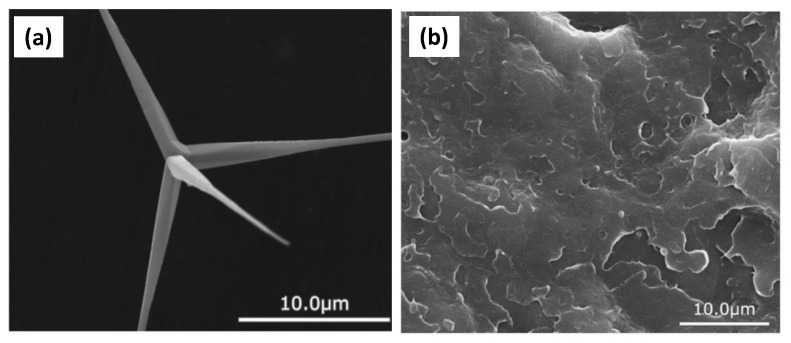
Typical SEM microphotograph of (**a**) a T-ZnOw whisker and (**b**) impact-fractured surfaces of composites with untreated T-ZnOw. Images from [20].

Like UHA fillers, the tetrapod-like ZnO whiskers(T-ZnOw) possess the same perfect surface as the traditional whiskers. Unlike traditional whiskers, T-ZnOw shows a unique three-dimensional structure with four needles growing from one point, with the angle between two needles being 109.28° [97]. As shown in Figure 10, [20] this special structure provides T-ZnOw -filled DRCs with is tropic rather than anisotropic properties, meaning forces can be more evenly distributed inside the T-ZnOw- filled DRCs. The fillers’ needles can create a more powerful interface through increased integration with the resin matrix. 

A study on tensile strength showed that T-ZnOw-reinforced DRCs could reach 26.5 MPa even if the T-ZnOw fillers were not treated with a coupling agent. The storage modulus was improved by adding T-ZnOw fillers. Moreover, the surface treatment of T-ZnOw fillers resulted in their becoming flexible. Either a silane coupling agent or a titanate coupling agent could improve the reinforcing effect of T-ZnOw fillers on the final composites. Though silane coupling agent treatment resulted in improved tensile strength, while titanate coupling agent treatment improved the impact strength [21], microcracks were formed at the interface between fillers and the resin matrix during the crosslinking process, leading to a change in the fracture mode of DRCs. This phenomenon was pronounced when the filler content was more than 10 wt%.

### 5.6. Core-Sheath Fillers

**Figure 11:**
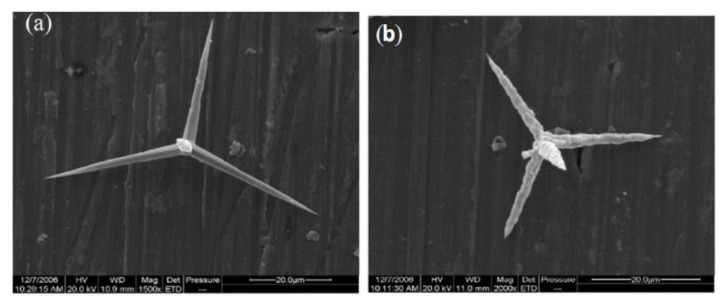
SEM micrographs of (**a**) an original T-ZnOw and (**b**) aT-ZnOw/PANI core-sheath structure. Images from [22].

In a study by Chen et al., a core-sheath structured T-ZnOw/polyaniline (PANI) composite filler was developed via graft polymerization [22]. As shown in Figure 11, PANI underwent in situ polymerization, which changed the surface wetting properties of T-ZnOw from hydrophilic to hydrophobic and, thus, facilitates interaction with the dental resin matrix; γ-aminopropyltriethoxysilane (APTS) played an essential role in ensuring a strong bonding and inducing in situ polymerization on the filler’s surface. At the same time, the core-sheath structured T-ZnOw/PANIhad an increased surface area and interfacial bonding, which endowed the composites with better performance.

**Figure 12:**
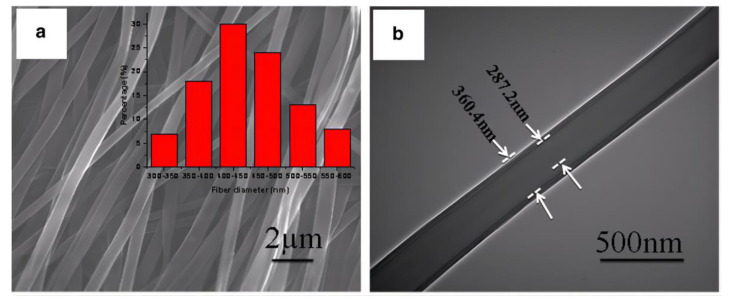
(**a**) SEM- and (**b**)TEM-micrographs of PAN-PMMA core-shell nanofibers. Images from [23].

Similarly, by examining the microstructure of the polymer-fiber-reinforced resin composite, Deng et al. [24] found that the bonding between polymer fibers and the resin matrix was not perfect. To improve the surface wetting and interfacial bonding strength, an electrospinning technique was used to fabricate core-sheath polymer nanofibers with polyacrylonitrile (PAN) as a core and poly (methyl methacrylate) (PMMA) as a sheath (Figure 12) [23]. Compared with pure resin, the flexural strength, elastic Young’s modulus, and fracture work of composites reinforced with 7.5 wt% PAN-PMMA nanofibers increased by 18.7%, 14.1%, and 64.8%, respectively. In addition, the PAN-PMMA nanofibers had a drug-eluting capability.

**Figure 13:**
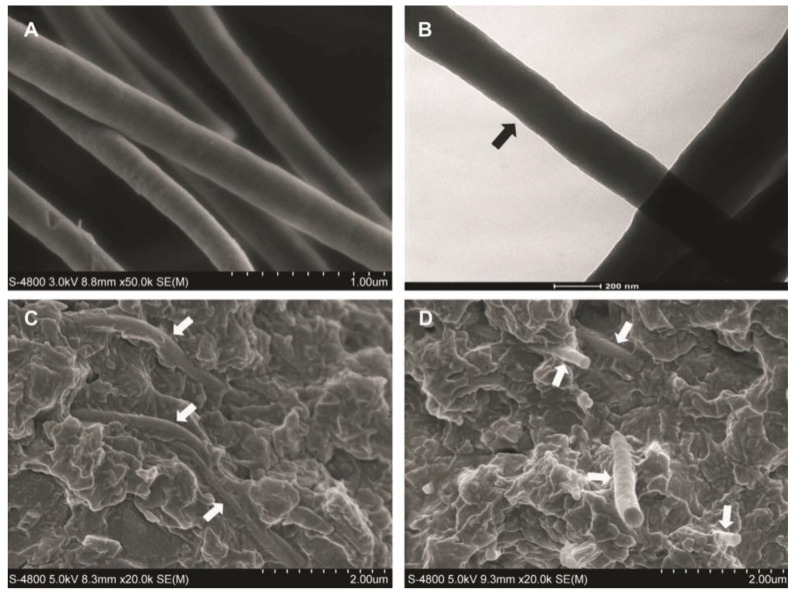
Morphology of electrospun ZY@S nanofibers: (**A**) SEM image (fiber diameter: 260 ± 39 nm) and (**B**) TEM image showing the core-sheath structure. Morphology of the composite fracture surface with (**C**) 2.5 wt% ZY@S nanofibers and (**D**) 5.0 wt% ZY@S nanofibers.Images from [98].

Likewise, Li et al. [98] prepared ceramic nanofibers with yttria-stabilized zirconia as a core and silica as a sheath (i.e., ZY@S nanofiber) via a reactive coaxial electrospinning process (Figure 13). Compared to the ZY fiber, the ZY@S nanofiber combined the strong and tough ZY ceramic nanofibers with a silica sheath, which enabled improved interfacial bonding via silanization, as used for glass fibers. Compared with the unloaded resin, the flexural strength of the resin composite filled with 2.5 wt% ZY@S ceramic nanofibers was significantly improved (45%).

### 5.7. Glass Flakes

Glass flakes are generally modified C glass fillers with a high aspect ratio. Commercial glass flakes are available in thickness ranging from 100 nm to 7 μm, with the d50 (median particle size) diameter ranging from 15 to110 μm, and are categorized into three nominal diameter distributions (unmilled, milled, and micronized). Glass flakes with excellent inherent strength and high surface area are fundamental in providing dimensional and thermal stability to DRCs [99].

Glass flakes can be used alone or as a reinforced filler with silica particles in the resin matrix for improving the mechanical properties of DRCs. Significant improvement in the hardness and compressive strength was reported by Motohiro et al. for glass-flake-reinforced DRCs. The improvement in the mechanical properties was dependent on the glass flake content and surface salinization conditions [100]. Glass flakes have also been shown to enhance the mechanical properties of silica-particle-filled DRCs, with γ-MPS salinization [101].

**Figure 14:**
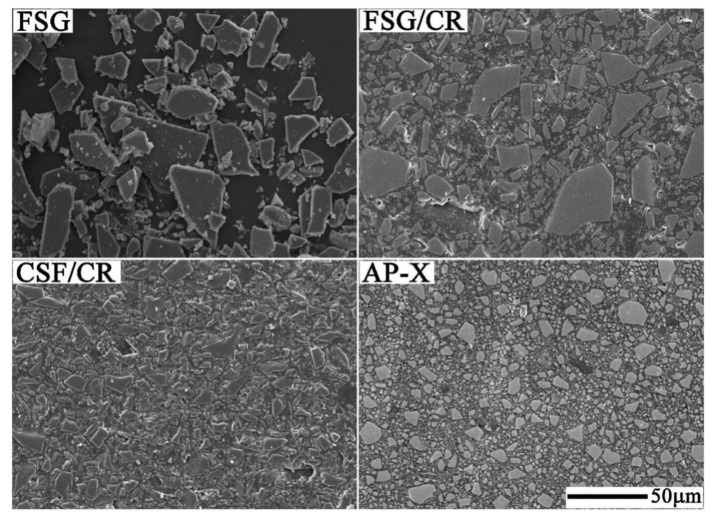
SEM images of flake-shaped glass (FSG), FSG/composite resin (CR) (60 wt%), crushed silica filler (CSF)/CR (60 wt%), and a commercial CR clearfil-AP-X. The FSG is platelet-shaped with a flat surface and is smaller than 50 μm. The particle size of FSG is larger than that of the CSF and the filler contained in AP-X. Images from [100].

Compared with other fillers, glass flakes have the additional advantage of improving the aesthetic property of DRCs. In a study by Motohiro et al., a glass-flake-filled composite resin showed better translucency compared to its irregularly shaped counterpart. This was attributed to the large particle size and flat surface of the glass flake, which suppressed light scattering (Figure 14) [100]. Though an increasing haze was presented with the addition of microglass flakes (MGFs), MGFs with a smaller aspect ratio showed a significantly lower haze compared to irregular glass particles. Moreover, refractive indices (RIs) of such composites could be adjusted by the addition of different amounts of softener. As a result, the residual haze could be as low as 2%, indicating high transparency [102].

With the concept of biomimetics, structural biological composites such as nacre (the protective inner layer of mollusk shells) offer much inspiration for the fabrication of composites with both superior mechanical properties and optical characteristics. Amini et al. combined glass flakes and poly (methyl methacrylate) (PMMA) using a centrifuge-based fabrication method that aligned and compacted the flakes into layers. This nacreous composite showed a four-fold increase in fracture toughness and a three-fold increase in flexural strength compared to conventional structural glasses. Additionally, by matching the refractive indices of the PMMA, a 73% average optical transmittance was achieved [103]. This novel feature has potential for application in DRC improvement.

### 5.8. Microcapsules

Microencapsulation is a novel approach developed for endowing DRCs with self-healing properties [104]. A microcapsule is a spherical particle with a polymer or glass shell that encloses healing liquid. When microcapsules are embedded into the resin matrix, they can rupture to release the healing liquid, which could flow into the cracked area and be exposed to a catalyst in the matrix and trigger polymerization to heal the crack [105,106].

Then et al. [107] and Wertzberger et al. [108] studied two traditional self-healing systems, urea–melamine–formaldehyde (UMF) encapsulated dicyclopentadiene (DCPD) (no catalyst) and DCPD microcapsules with Grubb’s catalyst, respectively. Their results showed that incorporation of a small number of microcapsules did not affect the performance of the resin matrix while the latter reported that 57% recovery of the original K_IC_ was achieved despite being substantially filled (55 wt%) with microcapsules. 

**Figure 15:**
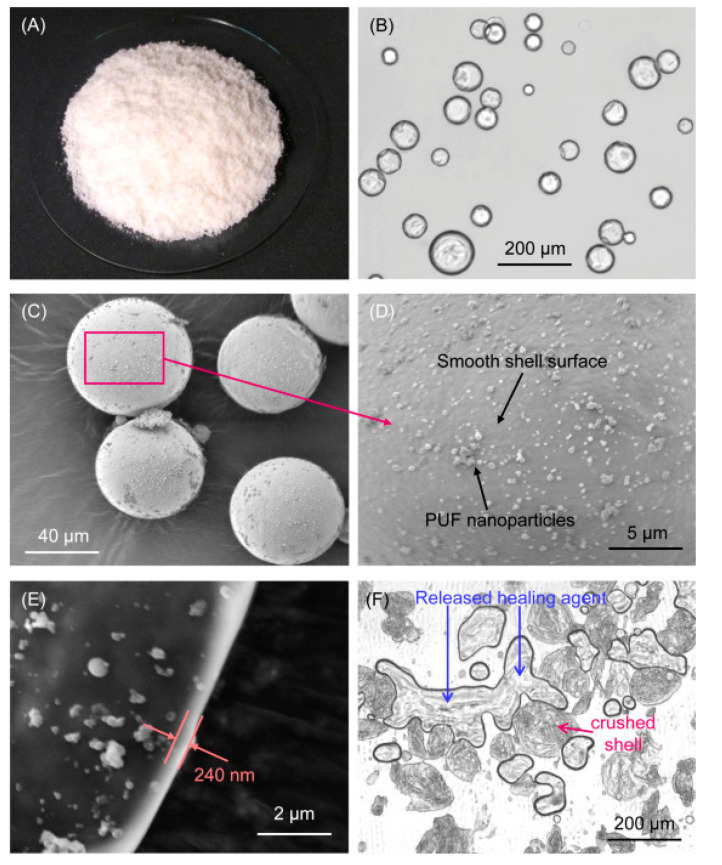
Microcapsules were prepared with a polymerizable TEGDMA–DHEPT healing liquid inside PUF shells. (**A**) Photo showing a pile of synthesized microcapsules. (**B**) Transmitting optical image showing the shell structure as a dark ring. (**C**) SEM image of typical microcapsules. (**D**) High-magnification SEM image of the shell surface showing nanoparticle deposits on an otherwise smooth shell surface. (**E**) High-magnification SEM image indicating the shell thickness. (**F**) Optical image of crushed microcapsules showing the released healing liquid films. Images from [111].

Considering the potential toxicity and ease of fracture when mixed with fillers of polyurethane microcapsules, novel self-healing dental composites (SHDCs) have been developed with the use of glass ionomer cement (GIC) and silicate microcapsules. They have two additional components: (1) a healing powder (HP), i.e., strontiumfluoroaluminosilicate particles, and (2) a healing liquid (HL), i.e., aqueous solutions of polyacrylic acids encapsulated in silica microcapsules [109,110]. Huyang et al. reported that the average healing efficiency reached up to 25% when 10 wt% microcapsules were added with a slightly reduced elastic modulus in SHDCs. [110] The morphological and chemical observations confirmed the healing procedure as fracture, deliver, and heal (Figure 15) [111]. Yahyazadehfar et al. tested the same self-healing system with different silane coupling agents under cyclic loading and showed that methacrylate silane (MA-silanized) SHDCs achieved the best balance of healing efficiency (24.2% ± 3.8%) and fracture toughness at 5 wt% microcapsule loadings. Moreover, a significant increase in the resistance to fatigue crack growth with an increase of 580% in the fatigue life was observed in this new material [109].

## 6. Conclusions and Perspectives

The emergence of new fillers has further enhanced the performance of DRCs. Compared to those used over the last decade, the new fillers show an improved bonding ability to the resin matrix. Moreover, the new fillers show a stronger ability for mechanical interlocking between the filler physical space and the resin matrix. This phenomenon is more prominent in porous materials, such as porous particles, mesoporous silica particles, and it is also present in other fillers with special shapes, such as UHA, fibrous fillers, and tetrapod-like whiskers. As well as in the improved chemical combination, core-sheath fillers have better bonding performance compared with the traditional filler. The shell with the external components will be adjusted according to the resin matrix chosen, such as glass vs. ZY@S, PAN vs. PAN-PMMA. In addition, the porosity of filler improves the drug-loading capacity, and the core-sheath structure enables the ability to control drug release.

In our search of review, the final physical properties of dental resin composites are greatly improved following the addition of morphology changed fillers. This improvement may be a result of the modified bonding capacity of the new fillers (e.g., the mechanical improvements of porous, mesoporous, and UHA fillers and the superiority of core-sheath fillers in chemical bonding). However, micro cracks still can be observed between the new morphology fillers and the resin matrix, some porous fillers still need more strength. Moreover, the core-sheath structure is a promising morphological feature, but preparation technology still needs to improve and innovate. It is these shortcomings that make the study of these fillers meaningful, which is also the significance of this work. Up to the present, we believe that the development of fillers may not be limited to the traditional morphology of granular and fibrous, or even the fusion of granular and fibrous types. New fillers, such as UHA and Tetrapod-like whiskers, may also be the focus of future research on fillers.

The ultimate goal of studying DRCs is to obtain a super excellent dental material for better oral service. At present, most of the current research on DRCs is focused on the matrix studying or strengthening comparison of several relatively single dental fillers, lacking comprehensive understanding, and longitudinal studies of the fillers. Moreover, interactions between two important components of DRCs, fillers, and matrix, are also of great importance. Hence, fillers need more historical reviews to get a new idea. The filler should be equivalent to the resin matrix, and fillers vs. resin matrix should be further considered. The interactions between fillers and the resin matrix should be continuously studied. Innovations in these areas are undisputedly the key to the development of truly high-performance DRCs to meet the ever-increasing future oral health needs.

## Figures and Tables

**Table 1:** Particulate fillers for dental resin composites ^a^.

Filler Types	Chemical Composition	Size (μm)	Shape	Commercial Composites
Ground quartz micropowder	SiO_2_	10–50	Irregular	Aelite Aesthetic Enamel (Bisco)
Ground glass micropowder	SiO_2_ + BaO + SrO_2_ + TiO_2_	0.6–10	Irregular	Admira (Voco) Artemis (Ivoclarvivadent)
Air colloid silica ultra-fine nanopowder	SiO_2_	0.04–0.4	Spherical	Enamel Plus HFO (Micerium) Fitek Supreme (3M ESPE)
Hydroxyapatite particle (HA)	Ca_5_(PO_4_)_3_(OH)	N.I. ^b^	Irregular	N.I.
Prepolymerization particle	SiO_2_ + resin matrix	0.04 + (0.6–1.0)	Spherical + irregular	Clearfil Majesty (Kuraray) TerticEvoCeram (Ivoclar North America)

^a^ All dental resin composites listed here include hybrid fillers instead of single fillers. ^b^ N.I. = Not informed. Information regarding the commercial composite has not been found.

**Table 2:** Characteristics of various fibrous fibers.

Filler Types	Chemical Composition	Dimension
Microwhisker	Silicon nitride Silicon carbide	Diameter: 0.1–2 μm; mean = 0.4 μm Length: 2–30 μm; mean = 5 μm Diameter: 0.1–3 μm; mean = 0.7 μm Length: 2–100 μm; mean = 14 μm
Short glass microfiber	Silica	Diameter: 10–17 μm Length: 14–2400 μm
Ceramic nanofiber	Silica, zirconia, zirconia-silica zirconia-yttria-silica	Diameter: 160–390 nm Length: 5–10 μm
Polymer nanofiber	Nylon-6	Diameter: 100–900 mm
Nanotube	Carbon	Diameter: 50–100 nm

**Table 3:** Types of novel-shaped fillers by morphology modification.

Filler Types	Chemical Composition	Shape	Size
Porous particle	Glass-ceramic	Particle-like	2–4 μm
Mesoporous particle	Silica	N.I. ^a^	Average 496 nm
UHA particle	Hydroxyapatite	Sea urchin	2–3 µm
Nanocluster particle	SiO_2_, ZrO_2_	Particlelike	0.07–2.7 µm
T-ZnO whisker	ZnO	Tetra-needle	0.18–0.21 μm
Core–sheath fiber	Zirconia/Silica, PAN-PMMA, T-ZnOw/PANI	Fibrous	Diameter: 220–300 nm Diameter: 300–400 nm N.I.
Glass flake	SiO_2_, Al_2_O_3_, CaO, et al.	Flakes	Diameter: 15–160 μm
Microcapsule	Polymer shell with healing liquid inside	Capsules	Diameter: 10–300 μm

^a^ N.I. = Not informed.

## Data Availability

Data available in a publicly accessible repository. Data sharing not applicable to this article as no datasets were generated or analyzed during the current study.
